# The effect of classroom environment on literacy development

**DOI:** 10.1038/s41539-023-00157-y

**Published:** 2023-04-03

**Authors:** Gary Rance, Richard C. Dowell, Dani Tomlin

**Affiliations:** The University of Melbourne, Department of Audiology and Speech Pathology, Parkville, VIC Australia

**Keywords:** Education, Human behaviour

## Abstract

The physical characteristics of a child’s learning environment can affect health, wellbeing and educational progress. Here we investigate the effect of classroom setting on academic progress in 7–10-year-old students comparing reading development in “open-plan” (multiple class groups located within one physical space) and “enclosed-plan” (one class group per space) environments. All learning conditions (class group, teaching personnel, etc.) were held constant throughout, while physical environment was alternated term-by-term using a portable, sound-treated dividing wall. One hundred and ninety-six students underwent academic, cognitive and auditory assessment at baseline and 146 of these were available for repeat assessment at the completion of 3 school terms, allowing within-child changes across an academic year to be calculated. Reading fluency development (change in words read-per-minute) was greater for the enclosed-classroom phases (*P* < 0.001; 95%CI 3.7, 10.0) and the children who showed the greatest condition difference (i.e. slower rate of development in the open-plan) were those with the worst speech perception in noise and/or poorest attention skills. These findings highlight the important role classroom setting plays in the academic development of young students.

## Introduction

The move away from didactic teaching pedagogies for Primary School-aged children in the 1960s and 1970s led to the implementation of “open-plan” learning environments in schools around the world. The potential advantages of this classroom configuration (where multiple grades are located within a single physical space) are that they may create a less authoritarian environment^1^ and support a greater range of learning methodologies and group sizes^2,3^. High levels of background noise and lack of acoustic privacy (created by greater numbers of students engaged in a variety of activities) within the same physical space have, however, been consistently identified by teachers and students as undesirable aspects of open-plan settings^4^. Furthermore, studies of these classroom spaces over the past four decades have consistently suggested that intrusive noise from adjacent class bases reduce speech intelligibility and increase distraction^4^. These in turn, create a significant educational risk as children spend much of their school time (45–60%) actively listening^5^ and it is crucial that the classroom allows them to comfortably hear and understand both teachers and classmates.

Speech perception in the classroom is affected by a range of factors including room geometry, teacher voice characteristics, reverberation time and background noise. Of these, background noise exerts the greatest influence on intelligibility by masking and distorting the target signal^6^. Auditory masking essentially takes two forms (“energetic” and “informational”), both of which are relevant to the classroom environment^7^. Energetic masking (EM) occurs when the background noise corresponds in time and frequency content to the target, leading to an overlap of excitation in the auditory periphery^8^. This, in turn, reduces the audibility of the target rendering it unavailable for processing at higher levels. Energetic masking is a particular issue in open-plan settings as the level of ambient noise (resulting from movement of desks and chairs, computers etc) is directly related to the number of students within a physical space^9^. Informational masking (IM) is centred at higher levels in the auditory pathway and is the result of perceptual interference caused by meaningful noise sources such as speech^10^. It occurs as a consequence of either degraded “object formation” i.e. segregation of the target from extraneous speech (such as intrusive voices from a second class-base in an open-plan setting) or impaired “object selection” – where the listener is required to direct his/her attention to the target speech and while ignoring other voices^11^.

Background noise also affects non-auditory (cognitive) functions. Importantly, the nature or content of the competing signal plays a significant role in the degree of disruption. For example, studies with adults have consistently shown that serial-recall of visually presented items is impeded by “task-irrelevant” sounds such as single taker speech, or even meaningless speech sounds (for a review see Schlittmeier et al.^12^.) In children, this effect on short term memory is even more pronounced and has been attributed to two distinct mechanisms. Firstly, competing sounds that change over time may interfere with the ordering of remembered information. Secondly, irrelevant sounds may capture the listener’s attention if the signal is particularly salient such as significant words (e.g. a person’s name) or an unexpected sound (e.g. a slamming door)^13,14^. This latter mechanism is thought to be the more important in primary-school aged children who are particularly susceptible to sound-related distraction as a result of immature attention control processes^15^.

The presence of background noise can also have marked effects on the performance of academic tasks in school-aged children—especially when the masker involves meaningful speech. Klatte et al.^15^ reviewed a series of studies evaluating the deleterious effect of noise on academic tasks including reading, spelling and arithmetic and found that most demonstrated impairments when the masker was a meaningful noise. Particularly affected were activities involving reading and writing, where the competing speech was thought to engage semantic functions which directly compete with the semantic processes involved in the task^16^. As such, we might expect performance on these core academic tasks to be adversely affected in the open-plan classroom setting, where irrelevant (but meaningful) speech from multiple class bases are a common occurrence.

Chronic exposure to high levels of background noise affects all aspects of classroom performance. Negative correlations have been demonstrated between classroom noise levels and the development of cognitive skills such as attention, concentration and memory^4,17,18^ and, as a result, overall academic progress may be impacted. Shield and Dockrell^19^ for example, in their large-scale study of classroom noise in UK primary schools found that Standardized Assessment Test (SAT) failure rates for mathematics, literacy and science in children aged 7- and 11 years increased by ≈5% for every 2 dB increase in classroom noise. Similarly, Puglisi et al. (2018)^20^ found a correlation between classroom acoustics and reading speed in normally developing 7–8 year-old students. Reading acquisition seems to be particularly susceptible to sustained noise exposure^14,21,22^ which may be a reflection of the fact that both speech perception and short-term memory are adversely affected by background noise and both play important roles in reading acquisition.

Despite the recent proliferation of open-plan classrooms there has been little research exploring the efficacy of these environments as learning spaces. In the current longitudinal study we measured within-child changes in reading fluency development as a function of classroom environment (open- vs enclosed-plan). Reading fluency was selected as primary outcome measure as we considered it likely to be affected by the sub-optimal acoustic characteristics of the open-plan setting and because it has been shown to be reflective of overall academic progress^23,24^. We also evaluated a range of cognitive and listening abilities to explore which learner characteristics might predispose a child towards a particular classroom setting.

## Results

### Reading ability at baseline

Baseline reading ability across the whole cohort was normal. Mean fluency rate was 111.3 (SD = 39.6) words per minute which is consistent with published norms and 9/196 participants (4.6%) showed WARP scores outside age-corrected normative values^25^.

Correlations were calculated between baseline reading scores and participant demographic data, audiometric and cognitive assessments. Reading scores were significantly associated with IQ (*r* = 0.261, *p* < 0.001), attention scores (*r* = 0.272, *p* < 0.001), and working memory scores (*r* = 0.219, *p* < 0.01). Baseline reading scores were not significantly correlated with participant age (*p* = 0.910) nor with speech recognition in noise ability (*p* = 0.709). There were significant correlations across the three cognitive assessments (Table 1).

**Table 1:** Pairwise Pearson Correlations showing the associations between baseline measures.

Measure 1	Measure 2	*N*	Correlation	95% CI for ρ	*P*-Value
Age	WARP	195	0.008	(−0.133, 0.149)	0.910
TONI-4	WARP	192	0.261	(0.123, 0.388)	0.000
Attention Quotient	WARP	192	0.272	(0.136, 0.399)	0.000
Digit Span	WARP	188	0.219	(0.079, 0.351)	0.002
LiSN-S	WARP	190	0.027	(−0.116, 0.169)	0.709
TONI-4	Age	193	−0.197	(−0.329, −0.057)	0.006
Attention Quotient	Age	193	−0.107	(−0.245, 0.035)	0.138
Digit Span	Age	189	−0.109	(−0.248, 0.035)	0.137
LiSN-S	Age	191	0.109	(−0.033, 0.248)	0.132
Attention Quotient	TONI-4	190	0.200	(0.059, 0.333)	0.006
Digit Span	TONI-4	186	0.317	(0.181, 0.441)	0.000
LiSN-S	TONI-4	188	−0.022	(−0.165, 0.121)	0.764
Digit Span	Attention Quotient	187	0.242	(0.102, 0.372)	0.001
LiSN-S	Attention Quotient	189	0.177	(0.036, 0.312)	0.015
LiSN-S	Digit Span	185	−0.021	(−0.165, 0.124)	0.776

**Fig. 1:**
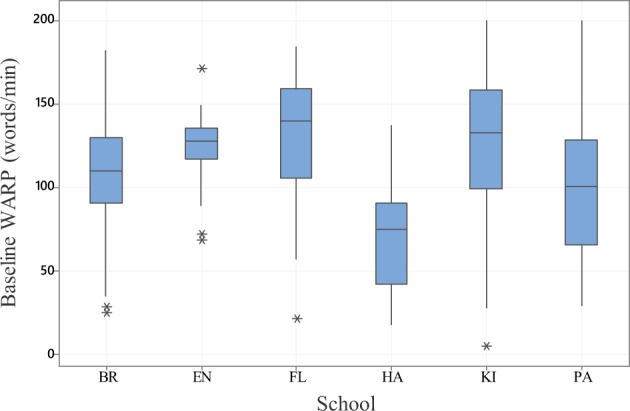
Baseline reading fluency (words per minute) for each school site. The centre line of each boxplot represents the data median and the bounds of the box show the interquartile range. The whiskers represent the bottom 25% and top 25% of the data range—excluding outliers which are represented by an asterisk.

The significant variables and “School” were combined in a general linear model to ascertain the independent predictors of baseline reading scores. This showed significant results for School (*F* = 4.93, *p* < 0.001), IQ (*F* = 6.72, *p* < 0.05) and Attention score (*F* = 7.47, *p* < 0.01). Working memory score was not a significant independent predictor of reading ability in this analysis. Tukey post hoc comparisons of the scores for different schools showed that HA had a significantly lower mean reading score than FL, KI, and EN (Fig. 1). School FL also had a significantly better mean reading score than BR and PA.

### Reading fluency across the data collection period

A mixed effect analysis was undertaken for all children who completed baseline, open- and enclosed classroom reading assessments. Individual participants were considered as a random variable with fixed factors of classroom condition, order of assessment (enclosed first or open first), and year of assessment. Note that “School” could not be included in this analysis as it is confounded with “year” and “order”. That is, each participant group in a particular school and year had the same order of testing. IQ and attention scores were included as covariates based on the initial analysis of baseline reading scores. The condition factor was significant (*F* = 108.3, *p* < 0.001) but “order” (*F* = 0.28, *p* = 0.597) and “year” (*F* = 1.50, *p* = 0.215) were not. IQ (*F* = 16.2, *p* < 0.001) and attention score (*F* = 20.76, *p* < 0.001) were both highly significant.

Tukey post hoc comparisons for the condition factor showed that the mean reading scores for open classroom (*M* = 128.3, *p* < 0.001) and enclosed classroom (*M* = 132.1, *p* < 0.001) were significantly higher than the baseline measurement (*M* = 111.3), indicating general improvement in reading fluency over the course of the study. The mean for enclosed classroom assessments was significantly higher than for open classroom (*p* < 0.05) in this analysis. The effect size for the difference between enclosed and open classroom scores based on the pooled standard deviation (*S* = 14.3) of the mixed effect model was 0.26 (weak).

**Fig. 2:**
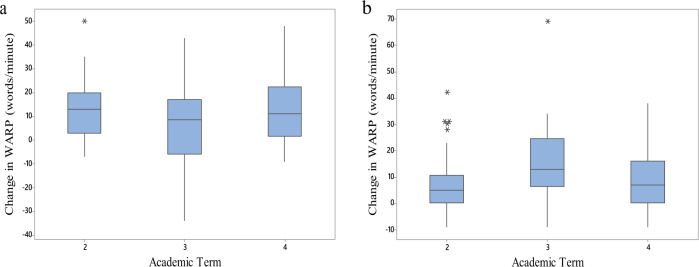
Change in reading fluency (term-by-term) for schools following each condition sequence. Panel (**a**) shows classes following the Enclosed/Open/Enclosed sequence and Panel (b) shows classes following the Open/Enclosed/Open sequence. The centre line of each boxplot represents the data median and the bounds of the box show the interquartile range. The whiskers represent the bottom 25% and top 25% of the data range—excluding outliers which are represented by an asterisk.

Analysis of within-child changes across classroom conditions indicated higher rates of reading development during the enclosed study phases. Mean change in WARP score for enclosed school terms was 14.0 (SD = 12.4) words/min and for the open-plan terms was 7.2 (SD = 12.9) words/min (*t* = 4.24, *p* < 0.001; 95% CI for paired difference: 3.7, 10.0 words/min). This difference is reflected in Fig. 2 which shows mean change-in-WARP scores calculated term-by-term for schools following a “Closed/Open/Closed” condition sequence (Panel a) and schools following an “Open/Closed/Open” (Panel B) schedule.

### Factors affecting reading fluency development

A single score reflecting the reading development bias towards one or other of the classroom conditions was calculated for each student who completed the study protocol. This value, termed the “environment score”, was the difference between reading development rate for the enclosed and the open classroom conditions and was determined as follows:

$${{{\mathbf{Environment}}}}\,{{{\mathbf{Score}}}}\,\left[ {{{{\mathbf{ES}}}}} \right] = {{{\mathbf{\Delta }}}}{{{\mathbf{WARP}}}}\,\left( {{{{\mathbf{enclosed}}}} - {{{\mathbf{plan}}}}} \right) - {{{\mathbf{\Delta }}}}{{{\mathbf{WARP}}}}\,\left( {{{{\mathbf{open}}}} - {{{\mathbf{plan}}}}} \right)$$

**Fig. 3:**
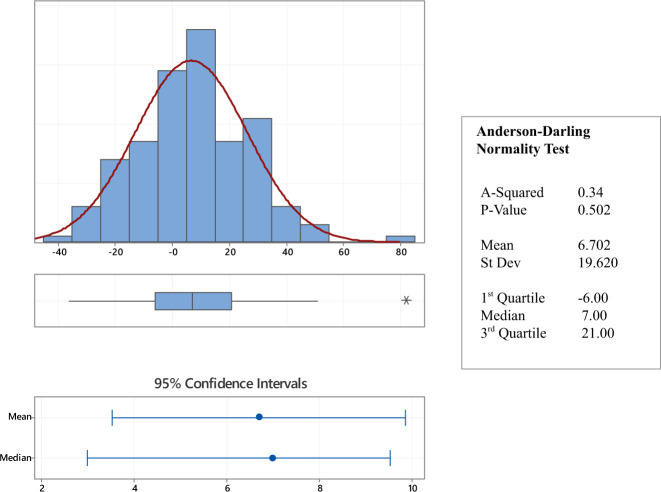
Distribution of Environment Scores for the 146 participants who completed the longitudinal study protocol. The centre line of the boxplot represents the data median and the bounds of the box show the interquartile range. The whiskers represent the bottom 25% and top 25% of the data range—excluding outliers which are represented by an asterisk.

Ninety-four of the 146 participants (64.4%) showed a positive ES indicating a higher rate of reading fluency development in the enclosed-plan study phases. Fig. 3 shows the distribution of ES values. The data were normally distributed with mean (6.70 words per minute) and median (7.0 words per minute) significantly above zero, that is, the enclosed classroom reading scores were significantly higher than for open classroom across the study. The effect size for the improvement in reading fluency for enclosed compared with open classroom, based on the standard deviation of the distribution (19.6) is 0.34. This is slightly different to the effect size derived from the mixed effects model, as that model includes all data whereas the ES distribution includes one score for each child completing the study protocol.

**Fig. 4:**
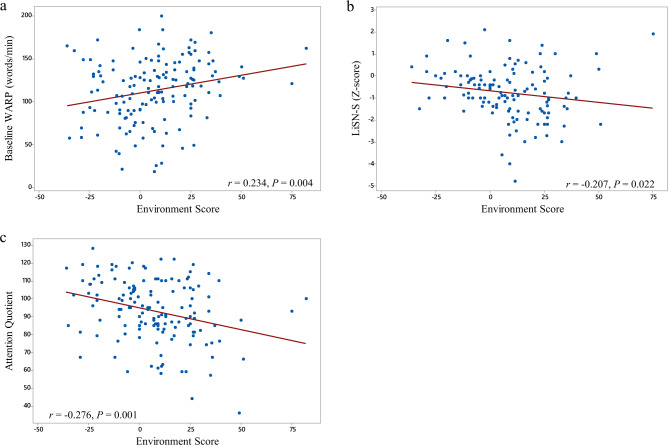
Reading fluency Environment Score (ES) for each student plotted as a function of participant characteristics. Panel (**a**) shows ES versus baseline reading fluency, Panel (**b**) shows ES versus speech perception in noise and Panel (**c**) shows ES versus Attention.

**Fig. 5:**
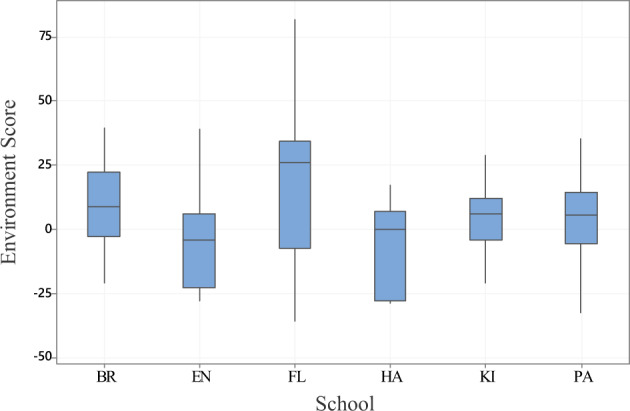
Environment Score (words per minute) for each school site. The centre line of each boxplot represents the data median, and the bounds of the box show the interquartile range. The whiskers represent the bottom 25% and top 25% of the data range.

A general linear model analysis with ES for each participant as the dependent variable was undertaken including School, baseline reading score, order of testing, age, IQ, attention scores, working memory, and speech recognition in noise scores as independent variables. Order of testing, age, working memory and IQ were not significant in the analysis. School (*F* = 3.24, *p* < 0.01), Baseline reading score (*F* = 5.33, *p* < 0.05), attention score (*F* = 10.52, *p* < 0.01), and speech recognition in noise (*F* = 4.92, *p* < 0.05) were significant independent predictors of the environment score (Fig. 4). Fig. 5 shows ES differences across schools. Results were broadly similar across sites, although Tukey post hoc comparisons did indicate a significant difference between school FL (which had the highest Environment Score) and the three schools with the lowest Environment Scores—PA, EN and HA.

## Discussion

In this study, we explored the potential impact of learning environment on academic progress comparing the effect of open- and enclosed-plan classrooms on normally developing children aged 7–10 years. Overall, reading fluency development was greater in the enclosed classroom and the children who showed the greatest environment effect (i.e. bias towards the enclosed classroom) were those with the poorest attention and listening skills.

A child’s rate of academic progress is influenced by a range of factors, some of which are inherent “learner characteristics” and some environmental. Consistent with the literature, baseline reading fluency in our cohort was correlated with a number of intrinsic, cognitive features including non-verbal IQ, working memory and attention^24,26,27^. These factors were, however, highly correlated with each other, and contrary to previous studies, working memory was not a significant independent predictor of reading ability. Listening capacity also showed no relation to baseline reading fluency, suggesting that the participants’ ability to perceive speech in the presence of background noise had not been a factor in overall literacy development prior to the study.

Baseline reading ability varied across the school sites. This was likely associated with socio-economic factors as the two schools with the lowest mean baseline WARP scores (HA and PA) were in regional locations and had the lowest levels of socio-educational advantage (Table 2). It is well established that on average, a student attending a school with lower peer socioeconomic status (SES) will show poorer educational outcomes (including reading) than one attending a school with a higher SES^28,29^.

**Table 2:** Site and study participation details for each of the collaborating schools.

School	Year	Socio-educational Advantage (ICSEA) School Percentile	Noise L_Aeq,(10 min)_ (dB)		Reverberation (s)		Condition Sequence	Participants (Baseline) Total: 196	Participants (completing open & enclosed terms) Total: 146	Age Range (years)
			Open	Enclosed	Open	Enclosed				
BR	2016	94	61.7	60.3	0.36	0.35	O/E/O	26	22	7.6–9.0
BR	2017	94	*	*	*	*	E/O/E	23	19	7.0–8.9
BR	2019	93	*	*	*	*	O/E/O	31	10	8.2–9.0
KI	2018	61	55.7	50.2	0.52	0.37	O/E/O	16	12	8.0–10.0
KI	2019	61	*	*	*	*	E/O/E	8	4	8.2–9.8
FL	2016	90	67.0	65.0	0.62	0.62	E/O/E	27	26	7.9–10.00
EN	2018	90	61.8	55.5	0.44	0.36	E/O/E	19	17	8.0–9.0
HA	2019	48	66.5	54.0	0.47	0.43	E/O/E	12	7	7.2–10.00
PA	2019	43	59.8	55.2	0.38	0.39	O/E/O	34	29	8.2–10.4

ICSEA: The Index of Community Socio-Educational Advantage (ICSEA) scale represents a school’s level of educational advantage and is primarily based on the family backgrounds of enroled students. See the My School website for details: http://www.myschool.edu.au.

Noise Level: L_Aeq,(10 min)_: Equivalent continuous sound level (A-weighted) recorded over a 10 minute sample period.

Condition Sequence: O/E/O Term 2: Open-plan; Term 3: Enclosed-plan; Term 4: Open-plan.

E/O/E Term 2: Enclosed-plan; Term 3: Open-plan; Term 4: Enclosed-plan.

Classroom configuration had a significant effect on rate of literacy development. Day-to-day teaching pedagogies were not prescribed as part of the study, but as many environmental factors as possible (teaching staff, class groups, curricula etc) were held constant through the test period while the only change to the physical classroom environment was the term-by-term deployment of the portable, sound-treated dividing wall. Manipulation of this single variable was associated with clear differences in academic progress with 64% of students showing a higher rate of reading fluency development in the enclosed-classroom condition. Mean **Δ**WARP fluency score was 6.8 words/min lower for each school term spent in the open-plan condition. When extrapolated across a whole year this corresponds to a 27 word/min delay which is approaching a 1 standard deviation difference in overall reading performance for children in this age group^25^. What the long-term impact of delays of this order may be, and whether they would resolve spontanteously after a period in a more conducive learning environment is unclear, but it is well established that reading and academic deficits in primary school can persist into adolescence/adulthood and can cause psychosocial and behavioural issues as children become disengaged at school^30,31^.

The masking effect of increased noise is one possible explanation for diminished reading fluency development in the open-plan classroom configuration. Average background noise levels (recorded with class groups engaged in a range of quiet learning activities), were broadly similar to those reported previously for open-plan classrooms^4^ and were higher (5.4 dB) than for the enclosed-plan configuration. In normally developing 7–10 year old children, a noise level difference of this order typically represents a decline in classroom speech intelligibility of ≈10–15%^32^ raising the possibility that students would require a significantly higher degree of listening effort to hear and understand what is said in this more challenging acoustic environment^33^.

In addition to the level of background noise, the type of noise present in the open-plan classroom is likely to impede speech understanding and communication. Previous studies have reported high levels of disturbance and distraction in open settings even when background noise levels have been relatively low^4^, suggesting that the “informational masking” effects of meaningful noise (i.e. student and teacher voices from other class bases) limit how well a child can hear their own teacher^4,19^. Furthermore, visual distraction from movement in adjacent classes is also thought to affect a child’s ability to understand speech in the open-plan setting^19^.

The link between more challenging listening conditions in the open-plan classroom and restricted academic development may, in part, be explained by the theory of cognitive resource allocation. This theory proposes that a finite, interactive pool of cognitive resources, including memory and attention, are flexibly allocated to an activity depending on the demands of the task. If these resources are channelled elsewhere, task failure may occur. When an incoming auditory signal is masked or degraded, the listener can compensate, filling in the perceptual gaps with knowledge and context^34^. The greater the signal degradation, the greater the shift to predominantly top-down (knowledge based) listening to compensate and the greater the cognitive load^35^. Listening effort and resulting fatigue has been demonstrated in primary school children in typical classroom conditions^36^. Comprehension in such circumstances may be restricted if the resource limits are exceeded as the demands of auditory processing become more effortful^37^. Results of the current study provide support for this theory with students demonstrating the poorest listening in noise skills tending to be those with the greatest negative academic impact in the (noisier) open-plan study phases. i.e. with poorer access to the speech signal requiring creating greater listening effort in the classroom.

The lack of significant interaction between working memory and reading development in the different classroom environments suggests that more than cognitive resource limitations may underly the observed effect. An alternate explanation to listening effort needs to be considered. Although working memory was not a predictive factor, attention capacity was strongly correlated with academic performance bias. Students with the weakest attention showed relatively slower progress in the open-classroom setting. Whilst the masking effect of meaningful noise has been shown to disrupt short-term memory and auditory tasks (which aligns with cognitive resource theory) a recent review by Klatte and colleagues^15^, has shown that meaningful noise can also impact non-auditory tasks such as reading. This phenomenon has been termed the Irrelevant Sound Effect (ISE). The ISE has been proposed to be due to an increased attention burden when trying to ignore the competing signal^15^. The ISE effect of noise on non-auditory performance is greater the younger the children are, with an age effect proposed as further support for the influence of attention in the tasks as younger children have less attentional control. This effect of noise on non—auditory task performance is not reduced when non-meaningful sounds are utilised, further supporting the theory that it is disruption to attention that impacts learning.

The increased attention burden due to meaningful noise creates an increase in cognitive, rather than listening effort. This increased cognitive effort to supress the distraction in turn creates additional working memory load and thereby impacting on the learning occurring^38^. The ISE cognitive effort theory aligns with results of this study, in so far as children with poorer attention skills (and therefore at greater risk from the increased burden on attention) experienced the greatest learning impact.

Overall reading ability (baseline fluency score) was also a factor in classroom environment preference, with good readers typically showing greater reading development in the enclosed-plan condition. This outcome is unexpected, given the positive correlation between baseline reading ability and attention and warrants further investigation.

The extent to which meaningful noise will impact an individual is determined by the unique combination of intrinsic factors the child brings into the classroom. This was borne out by the findings of the current study where participants were not equally affected by classroom environment. While most showed a performance bias towards the enclosed plan setting, some were unaffected by the change in physical environment and small proportion even showed a significantly higher rate of academic development in the open-plan classroom. This latter group (typically comprising students with superior listening skills and/or better command of attention) may have been relatively unaffected by the extra acoustic challenges posed by the open classroom, allowing them to benefit from the pedagogical flexibility afforded by the setting. Children with poorer speech-in-noise or attention skills were, however, found to be at increased risk of either spending more time disengaged from educational activities in the open-plan environment or requiring more cognitive resources to maintain attention leaving fewer to facilitate their learning.

There were a number of study limitations. A more detailed analysis of acoustic conditions in the different classroom settings may have provided specific insights into the impact of background noise on learning in the open- and enclosed classroom settings. For example, to minimise classroom intrusion we took 10 min noise samples during reasonably consistent classroom activities (i.e. group work with minimal movement) and found that background noise levels were higher in the open-plan configuration. Sound-level recordings over a longer time-period (perhaps 8 h) would have provided more accurate noise estimates, taking into account level fluctuations over the course of the entire school day. Similarly, the A-weighted sound measures (which filter low-frequency energy) used in this study are likely to have underestimated the levels of background noise present in each classroom condition. We used the same weighting in both open- and enclosed-classroom recordings so the relative difference may not have been affected, but it is possible that one classroom condition had more low-frequency noise than the other. This is potentially important as low-frequency energy plays a critical role in listening effort and fatigue. As such, adding a measurement with the more linear dB(C) weighting could provide extra information about the degree to which low-frequency noise is an issue in different settings.

The findings of this study suggest a link between increased listening effort in the noisier/more distracting open-plan setting and the development of reading fluency. The present work cannot, however, be taken as proof of this relationship as there were no direct measures of listening effort. Future studies might include behavioural (response time on psychophysical tasks) and/or physiologic (pupil dilation) measures as evidence of a causal relationship^36,39^.

This study only considered reading fluency as a measure of academic progress and other aspects of learning development may be unaffected (or even augmented) by the open-plan classroom configuration. WARP reading fluency scores have, however, been strongly correlated in Australian students with each of the reading, writing, spelling, grammar and numeracy metrics from the National Assessment Program of Literacy and Numeracy (NAPLAN) assessment, suggesting that WARP findings are a strong indicator of overall academic progress^24^.

Only children 7–10 years were enroled in the study and the data cannot be directly extrapolated to other age groups. It is, however, likely that younger students whose auditory neural systems are still developing and whose lower levels of linguistic knowledge would restrict their ability to compensate for missing information^32,40–43^, would show even greater energetic and informational masking effects and greater learning consequences in the open-plan classroom. Furthermore, cognitive skill development occurs across childhood with the steepest rate of development between seven and nine years of age^44–46^. Younger students as a group, are therefore less likely to have the requisite cognitive resource pool to navigate the increased listening and attention challenges posed by the open-plan learning environment.

Participants in this study were all audiometrically normal throughout the data collection period and had no known cognitive or learning difficulties. Groups of children who are particularly vulnerable to the effects of noise on speech understanding including those who are hearing impaired^47^, those with auditory processing difficulties, those with language/learning disorders and those who are non-native speakers^4,24^ are likely to show even greater learning delays in the open-plan classroom.

In summary, the results of this study highlight the important role classroom setting plays in the academic development of young students. Exposure to the open-plan classroom environment resulted in considerably slower rates of reading fluency development across the whole cohort and particularly in those children with relatively poor attention and/or speech in noise skills. This finding is likely associated with increased levels of background noise occurring as a result of higher student numbers and multiple class activities in the one physical space.

The results of this study further suggest that care must be taken if open-plan spaces continue to be utilised. Whilst positive learning and social development opportunities can be provided by open-plan classrooms, appropriate and adequate measures to facilitate speech access should be applied. These include acoustic treatment to maximise sound absorption of ceilings/wallsand lowered ceilings to optimise listening conditions^4,42^. Consideration should also be given to visual barriers or operable walls to minimise visual distractions. Careful intentional design of learning spaces to ensure that conditions are optimal for all students will likely have direct positive outcomes on the academic development of young students.

## Methods

### Ethical Approval

This study was approved by the Ethics Committee of the Royal Victorian Eye & Ear Hospital and by the Research in Victorian Schools and Early Intervention Services office, Melbourne Australia and conformed to the tenets of the Declaration of Helsinki. Participation was voluntary and written consent was obtained from each child’s parent/guardian prior to study commencement.

### Participating Schools

Data collection was carried out in 6 mainstream Primary Schools (four metropolitan and 2 regional) over a 4 year period (2016–2019) (Table 2). All of the schools were in residential areas with no local industrial activity. The teachers were asked to indicate if there had been any changes in the local environment (construction work, changes to aircraft flight paths etc) that had produced a noticeable change in environmental noise levels over the course of the study period. No changes were reported. Four sites participated for a single year, one for 2 years and one for 3 years. For schools participating over multiple calendar years, teaching staff and class locations were held constant, but different groups of students were evaluated each year. Schools were selected based on the availability of open-plan classrooms able to accommodate two separate class groups within a single physical space. As part of the study, each classroom was fit with a portable, dividing wall (HUFCOR Series 2700 Acoustic Accordion Door) allowing the space to be bisected. An “enclosed” environment could therefore be created with one class group on either side of the partition. Removal of the dividing wall created the “open plan” environment. The partition was sound-treated with a Weighted Sound Reduction Index (R_W_) rating of 27 dB.

The class groups participating in this study were typical of those in Government Schools across the State of Victoria. Average class size (July 2021) reported for Year 3–6 classes was 23.2 students, and approximately 30% of schools were using open-plan spaces with two (or more) discrete class groups (https://www.education.vic.gov.au/Documents/about/department/brochurejuly.pdf). In our study, individual class sizes ranged from 22 to 25 students. For the open-plan condition, two discrete class groups (each with their own teacher), were based in the same room which meant that at full attendance, between 44 and 50 children were physically located within the open-plan space. Over the course of each week in the open-plan condition there were some joint learning sessions (involving both teachers), but for the most part the two class groups worked independently—each managed by their own teacher. As such, when the class groups were separated for the enclosed classroom condition, there was no change to the teacher/student ratio.

### Classroom acoustics

Acoustic sampling was undertaken at each site in both open and enclosed configurations. The classrooms were occupied in both conditions and the children were engaged in group work (with talking allowed) but minimal movement. Samples were taken at approximately the same time of day (45 min into the morning session). Recordings were obtained from the centre of each room using a SVAN971 (Class 1) sound level metre. Ten-minute noise samples (recorded in dBA) were obtained for each classroom configuration. Reverberation time (RT) was determined using the integrated impulse response technique according to the ISO 3382 measurement standard. Reverberation time was defined as the time taken for the level of a brief, broad-band stimulus (hand-clap) to decay by 60 decibels [RT(60)] and was the average of recordings at octave frequencies from 125 Hz to 8 kHz. As the acoustic spectra generated by a clap is somewhat variable, we maintained a regular hand configuration (cupped and at an angle) to optimise the low-frequency spectrum and minimise inconsistency. Noise level and RT(60) findings for each test site are shown in Table 2. The rooms were typically well acoustically treated with carpeted floors, sound-absorbent pin-boards on walls and few exposed hard surfaces. As such, reverberation times were relatively low (i.e. within the range recommended for typically developing children)^48^ and showed no difference between open and enclosed classroom conditions (Enclosed: mean=0.42, SD = 0.09 s; Open: mean=0.45, SD = 0.09 s, Paired-T: *p* = 0.289, 95%CI: −0.03, 0.09). Background noise levels were also relatively low in the enclosed classroom condition, but showed a significant increase (5.4 dB) in the open-plan configuration (Enclosed: mean = 56.7, SD = 5.2 dB L_Aeq,_; Open: mean = 62.1, SD = 4.2 dB L_Aeq,_, Paired-T: *p* = 0.021, 95%CI: 1.20, 9.57). This difference is unlikely to have been the result of a change in the acoustic properties of the classrooms. Bisection of the space (by the accordion door) halves the volume of each room and splits the sound absorption area, but the overall sound power in each enclosed room is reduced as the number of students (the primary noise source) per classroom is also halved. As such, the noise level in open and enclosed conditions would be expected to be similar if only the physical properties of the spaces had changed. The reason for the measured difference (which was reasonably consistent across test sites [Table 2]), is unclear, but may reflect an increase in the activity noise made by students in the open-plan configuration. This phenomenon (known as the Lombard Effect) occurs when pupils feel they need to increase their vocal effort to both hear themselves and be heard in noisy situations. Increases in vocal output of approximately 6–7 dB have been reported for children in background noise levels equivalent to those observed for open-plan classrooms in this study (60–65 dBA)^49^.

### Study design and participants

Over the course of one academic year, room configuration (open versus enclosed) was alternated term by term. Condition order was randomised across schools for the first year of participation. For those schools who took part across multiple years, condition order was alternated year by year. Four student groups followed an Open/Enclosed/Open condition sequence across Terms 2, 3 and 4, and five groups followed an Enclosed/Open/Enclosed schedule (Table 2).

Each child whose class was located within the room(s) undergoing condition change was invited to participate in the study. Only those students whose parent/guardian consented to have them take part in the data collection were included. The participation rate (across all test sites) was approximately 45%.

One hundred and ninety-six students (88 girls) aged between 7.0 years and 10.4 years (mean=8.6, SD = 0.5 years) underwent baseline evaluation. A breakdown of participant age range for each school is shown in Table 2. One-hundred and forty-six children completed the longitudinal protocol allowing within-child comparison of development across open- and enclosed-plan study phases. Of the 146 evaluated across both open and enclosed terms, 73 were in classes undergoing the Open/Enclosed/Open schedule and 73 were in the Enclosed/Open/Enclosed (Table 2). All had normal sound detection levels (screened at 20 dBHL) for pure tones at octave frequencies between 250 Hz and 8 kHz. Each participant was considered by the primary classroom teacher to be typically developing and was enroled in Grade 3 or 4 at the time of the study.

Baseline data collection was undertaken at the beginning of Term 2 (3 months into the academic year [late March/early April]) and then repeated in the final week of Terms 2 (June), 3 (September) and 4 (December). Each school Term lasted 10 ± 1 weeks. Change values representing the difference in test score across each Term were determined. Where development across two terms with the same classroom condition was measured (eg. the Open-plan phases for class groups following Open/Enclosed/Open schedule) an average of the change values for the two terms was used.

For behavioural data collection each participant was removed from class and individually assessed in a quiet room with low levels of background noise (<40 dBA). Each child was evaluated one-on-one by an experienced study researcher.

### Materials

Primary academic outcome measure for the study was reading fluency which is a strong predictor of educational outcomes in primary school children^23,24^. Furthermore, reading fluency has been demonstrated to be influenced by the acoustic environment with poorer classroom signal-to-noise ratio correlated with poorer performance^20^. Reading fluency was assessed using the Wheldall Assessment of Reading Passages (WARP)^25^. Participants were required to read three × 200 word passages and an average number of words correctly read per minute was calculated. Reported performance ranges (mean ± SD) for participant ages represented in the study were as follows: 7 years: 84 ± 37 words/min; 8 years: 109 ± 40 words/min; 9 years: 118 ± 39 words/min and 10 years: 135 ± 39 words/min. The WARP has been shown to have both high parallel form reliability (0.94–0.96) and internal consistency (0.97 to 0.99)^50,51^.

A range of participant characteristics thought likely to impact reading development were also evaluated at baseline and at each subsequent data collection point to explore interactions between cognitive and listening variables. Reading fluency is a complex skill relying on the integration of various higher-level processes including attention and working memory^52,53^. Similarly links have been found between attention and working memory with performance on auditory listening tasks^24,54^.

General cognitive ability was assessed using the Test of Non-Verbal Intelligence (TONI-4)^55^. This task required the completion of 10 visual patterns using multiple choice options which increased in complexity. The results provide information about a child’s intelligence with minimal linguistic influence and are compared to age-specific normative data to produce normalised IQ scores. The TONI has demonstrated high test-retest reliability with high correlation coefficients (0.89) and limited random measurement error^56,57^.

Auditory working memory was evaluated using the Digit Span (reversed) subtest of the Clinical Evaluation of Language Fundamentals 4 (CELF-4)^58^. This test requires the repetition (in reverse order) of a series of numbers of increasing length and reflects auditory working memory, executive function and attentional control^59,60^. An age-corrected (scaled) digit span score was calculated for each child. Measures of reliability and validity of the CELF-4 are provided in the examiner’s manual with an internal consistency reliability coefficient of 0.78 and standard error of measurement of 1.41 provided.

Binaural speech perception ability was evaluated using the Listening in Spatialized Noise (LiSN-S) test. This assessment measures the participant’s capacity to segregate a target speech signal from competing speech noise^61^. The test stimuli (both target and noise) are administered under headphones, but a 3-dimensional auditory environment is created by synthesising the auditory signals with head-related transfer functions. Speech reception threshold ([SRT] signal-to-noise ratio required to identify 50% of the words in target sentences) was established for the DV90 listening configuration, where target speech and noise were different voices and presented from different directions. That is, the target signal was presented from 0^0^ azimuth while the competing speech was presented from a 90^0^ azimuth. Raw SRTs were age-corrected to produce a Z-score which was used in the analyses. This test has a demonstrated test-retest reliability coefficient of 0.7^62^.

Auditory and visual attention was assessed using the Integrated Visual and Auditory Continuous

Performance Test (IVA-CPT)^63^. Each child was presented with 500 trials of ‘1’s and ‘2’s in a pseudo-random pattern to assess sustained visual and auditory attention. Participants are required to click a computer mouse when the number “one” is seen or heard but to ignore any number “two” stimuli. The child’s scaled scores were calculated and compared with age and gender norms by the IVA-CPT software. An “Attention Quotient” based on both auditory and visual attention findings including measures of vigilance (omission errors), focus (variability in processing speed) and speed (reaction time) was used in the analyses. Average Attention Quotient score is 100 and the standard deviation 15. As reported in the Interpretation Manual, the IVA-CPT Attention Quotient has a test-retest *r* value of 0.74.

### Statistical analysis

Data were analysed using the MINITAB 19 statistical package. All assumptions for parametric analyses were met. Normality of data distribution was assessed using Anderson-Darling Normality tests. Correlations were calculated for baseline reading scores against participant demographic data, audiometric and cognitive assessments and the significant factors were included as independent variables in a general linear modelling analysis with baseline reading score as the dependent variable. Mixed effect linear modelling was used to analyse the complete data set. This analysis included participant as a random variable, timing (year), order and classroom condition as categorical factors and the significant cognitive and audiometric measures as covariates. Finally, a general linear model analysis was conducted on the Environment Scores (difference for each child between reading development scores in open and enclosed classrooms) including demographic, cognitive and audiometric measures and baseline reading score as independent variables. In all multivariate analyses Tukey post-hoc tests were used to assess pairwise significant differences for categorical variables where appropriate.

### Reporting summary

Further information on research design is available in the Nature Research Reporting Summary linked to this article.

## Supplementary information

Reporting Summary

## Data Availability

The data that support the findings of this study has been made available through the OSF Home data storage repository (Hyperlink: osf.io/5mn2b). Further information will be provided to suitably qualified researchers by the Corresponding Author upon request.
