# Supplementary material for: The effect of classroom environment on literacy development
Source: NPJ Sci Learn. 2023 Apr 3;8:9. doi: 10.1038/s41539-023-00157-y (PMC10070343; doi:10.1038/s41539-023-00157-y)
Supplement: Supplementary file 1 — Reporting Summary [file 41539_2023_157_MOESM1_ESM.pdf]

nature portfolio

Corresponding author(s): Gary Rance

Last updated by author(s): Mar 4, 2023

Reporting Summary

Nature Portfolio wishes to improve the reproducibility of the work that we publish. This form provides structure for consistency and transparency in reporting. For further information on Nature Portfolio policies, see our Editorial Policies and the Editorial Policy Checklist.

Statistics

For all statistical analyses, confirm that the following items are present in the figure legend, table legend, main text, or Methods section.

n/a	Confirmed	
☐	☒	The exact sample size (*n*) for each experimental group/condition, given as a discrete number and unit of measurement
☐	☒	A statement on whether measurements were taken from distinct samples or whether the same sample was measured repeatedly
☐	☒	The statistical test(s) used AND whether they are one- or two-sided<br>*Only common tests should be described solely by name; describe more complex techniques in the Methods section.*
☐	☒	A description of all covariates tested
☐	☒	A description of any assumptions or corrections, such as tests of normality and adjustment for multiple comparisons
☐	☒	A full description of the statistical parameters including central tendency (e.g. means) or other basic estimates (e.g. regression coefficient) AND variation (e.g. standard deviation) or associated estimates of uncertainty (e.g. confidence intervals)
☐	☒	For null hypothesis testing, the test statistic (e.g. *F*, *t*, *r*) with confidence intervals, effect sizes, degrees of freedom and *P* value noted<br>*Give P values as exact values whenever suitable.*
☒	☐	For Bayesian analysis, information on the choice of priors and Markov chain Monte Carlo settings
☒	☐	For hierarchical and complex designs, identification of the appropriate level for tests and full reporting of outcomes
☒	☐	Estimates of effect sizes (e.g. Cohen's *d*, Pearson's *r*), indicating how they were calculated

Our web collection on statistics for biologists contains articles on many of the points above.

Software and code

Policy information about availability of computer code

Data collection	N/A
Data analysis	N/A

For manuscripts utilizing custom algorithms or software that are central to the research but not yet described in published literature, software must be made available to editors and reviewers. We strongly encourage code deposition in a community repository (e.g. GitHub). See the Nature Portfolio guidelines for submitting code & software for further information.

Data

Policy information about availability of data

All manuscripts must include a data availability statement. This statement should provide the following information, where applicable:

- Accession codes, unique identifiers, or web links for publicly available datasets
- A description of any restrictions on data availability
- For clinical datasets or third party data, please ensure that the statement adheres to our policy

Provide your data availability statement here.

Human research participants

Policy information about studies involving human research participants and Sex and Gender in Research.

Reporting on sex and gender	Data was collected from both boys and girls.<br>No analyses were undertaken based on gender.
Population characteristics	196 normally developing children (88 girls) aged 7.0-10.4 years at assessment.
Recruitment	School-based study with every student enrolled in participating classes invited to take part. Only those children whose parents/guardians provided written consent were included. Participation rate was approximately 45%. No known biases likely to impact the results.
Ethics oversight	Approval from the Human Research and Ethics Committee of the Royal Victorian Eye & Ear Hospital

Note that full information on the approval of the study protocol must also be provided in the manuscript.

Field-specific reporting

Please select the one below that is the best fit for your research. If you are not sure, read the appropriate sections before making your selection.

☐ Life sciences ☒ Behavioural & social sciences ☐ Ecological, evolutionary & environmental sciences

For a reference copy of the document with all sections, see nature.com/documents/nr-reporting-summary-flat.pdf

Behavioural & social sciences study design

All studies must disclose on these points even when the disclosure is negative.

Study description	Investigation of the effects of classroom environment (open-plan vs enclosed plan) on reading fluency development in normally developing school children. Quantitative study comparing literacy development across school-terms as classroom condition (open-plan or enclosed plan) was alternated term-by-term.
Research sample	Normally developing Grade 3-4 children attending 6 participating elementary schools. 196 children (88 female) provided a respresentative sample.<br>Schools were selected on the basis that they had a suitable physical environment (open-plan classroom) for the study.
Sampling strategy	Convenience sampling. Quantitative data with sample size determined by power calculation (based on previously published data) to be sufficient.
Data collection	Behavioural assessment of academic, cognitive and speech perception abilities using standardized clinical measures. Data obtained one-on-one with each participant. The testing was carried out by an experienced research assistant who was blinded to study hypotheses. A range instruments with differing participant response and data recording types: transcribed verbal responses; participant computer input; pen and paper.
Timing	Longitudinal data collection with 4 x data collection points 10-12 weeks apart.
Data exclusions	no data excluded from the analysis
Non-participation	196 participants underwent baseline data collection and 146 completed the longitudinal protocol. No participants "dropped out". Rather they were not available on one of more occasions when school-based assessment was taking place. Typically due to illness.
Randomization	Classroom condition order was randomized across schools with half undertaking an enclosed-plan term first and the other half and open-plan term first. Each participant at a particular school followed the same schedule.

Reporting for specific materials, systems and methods

We require information from authors about some types of materials, experimental systems and methods used in many studies. Here, indicate whether each material, system or method listed is relevant to your study. If you are not sure if a list item applies to your research, read the appropriate section before selecting a response.

Materials & experimental systems

n/a	Involved in the study
☒	☐ Antibodies
☒	☐ Eukaryotic cell lines
☒	☐ Palaeontology and archaeology
☒	☐ Animals and other organisms
☒	☐ Clinical data
☒	☐ Dual use research of concern

Methods

n/a	Involved in the study
☒	☐ ChIP-seq
☒	☐ Flow cytometry
☒	☐ MRI-based neuroimaging
